# Effect of Plantar Sensory Stimulation on Sensorimotor Organization in General Joint Hypermobility: A Randomized Controlled Study

**DOI:** 10.3390/healthcare13202572

**Published:** 2025-10-13

**Authors:** Ramazan Yildiz, Ayse Yildiz, Onur Camli, Hüseyin Akkaya, Mehmet Aydin, Zekiye Basaran

**Affiliations:** 1 Department of Physical Therapy and Rehabilitation, Faculty of Health Sciences, Erzurum Technical University, Erzurum 25100, Turkey; ramazan.yildiz@erzurum.edu.tr (R.Y.); akkayahuseyin661@gmail.com (H.A.); mehmet.aydn4405@gmail.com (M.A.); 2 Department of Mathematics, Faculty of Science, Erzurum Technical University, Erzurum 25100, Turkey; onur.camli@erzurum.edu.tr; 3 Department of Physical Therapy and Rehabilitation, Faculty of Health Sciences, Gazi University, Ankara 06560, Turkey; zekiyesadbasaran@gmail.com

**Keywords:** general joint hypermobility, proprioception, sensory stimulation, randomized controlled study, mixed model ANOVA

## Abstract

**Background:** Individuals with generalized joint hypermobility (GJH) often exhibit altered sensorimotor control, which may contribute to balance and proprioception deficits. This study investigated the effects of sensory training applied to the plantar surface on sensorimotor organization components, including light touch, vibration, two-point discrimination, proprioception, muscle strength, and balance, in individuals with GJH. **Methods:** This randomized controlled trial included 65 asymptomatic individuals aged 18–25 years with a Beighton score of 5 or higher. The participants were randomly assigned to either the treatment (*n* = 32) or control (*n* = 33) group. The treatment group was given a 2-week home program that included plantar sensory training and an informative brochure on healthy foot care; the control group was given only the brochure. Light touch, two-point discrimination, vibration sense, proprioception, muscle strength, and balance parameters were assessed before and after the intervention. **Results:** Compared to the control group, the treatment group demonstrated significant improvements in light touch (*p* < 0.01), two-point discrimination (*p* < 0.01), vibration sense (*p* < 0.01), and proprioceptive accuracy (*p* < 0.01). Balance performance improved markedly in the posterolateral direction (+8.3 cm, *p* < 0.01), while anterior and posteromedial directions showed moderate but nonsignificant gains. Muscle strength showed no statistically significant changes across groups (*p* > 0.05). The control group exhibited no meaningful pre-post changes. **Conclusions:** Sensory training directed at the plantar surface results in positive changes in various components of sensorimotor organization in individuals with GJH.

## 1. Introduction

Generalized joint hypermobility (GJH) is characterized by an excessive range of motion in multiple joints and is commonly assessed using the Beighton scoring system [1,2]. The range of motion is examined using the Beighton scoring system, which ranges from 0 to 9 points, assessing five joints. Individuals who score five or above are diagnosed with GJH [1]. Epidemiological studies indicate that GJH is relatively common in the general population, with prevalence rates ranging from 10% to 30%, depending on age, sex, and assessment criteria [3,4]. Higher rates are observed in females, children, and adolescents, while specific populations, such as dancers and gymnasts, demonstrate even greater prevalence due to repetitive training and ligamentous laxity [5,6,7].

GJH is associated with musculoskeletal complaints, including arthralgia, subluxations, and osteoarthritis [8,9]. Although GJH is not officially classified as a pathological condition, it deserves careful investigation because its inadequate management may predispose individuals to recurrent injuries, reduced sport performance, and compromised quality of life [10,11,12]. Therefore, understanding effective intervention strategies in this population is essential for preventive and rehabilitative purposes. Accordingly, various interventions have been investigated in GJH, including resistance training [13], Pilates-based exercise [14], and proprioceptive programs [15]. However, results on balance and sensorimotor function are inconsistent. For instance, resistance training demonstrated limited effects on muscle properties in women with GJH [13], whereas a Pilates intervention for children improved pain and quality of life [14]. Furthermore, somatosensory-focused interventions have been shown to enhance proprioceptive accuracy in young dancers with GJH [15]. These studies focused on pain reduction, physical performance, or the effects of general exercise. To date, no randomized controlled trials (RCTs) have specifically examined the effects of plantar sensory training on sensorimotor organization in this population.

Sensorimotor organization refers to the central nervous system’s ability to perceive body position, integrate sensory information, and generate appropriate motor responses. The plantar surface, densely innervated with mechanoreceptors such as Merkel cells, Meissner’s corpuscles, Pacinian corpuscles, and Ruffini endings, plays a pivotal role in this process [16]. These receptors provide essential afferent feedback regarding pressure, vibration, and skin stretch, which is integrated with proprioceptive input from muscles and joints. Enhanced afferent input from the plantar surface can modulate cortical excitability, enlarge the cortical representation of the foot, and facilitate more efficient sensorimotor coupling [17,18]. Neurophysiological evidence indicates that such mechanisms contribute to anticipatory postural adjustments, reduced sway amplitude, and faster corrective responses [19,20].

In populations with impaired plantar sensation—such as older adults, individuals with diabetic neuropathy, and patients with multiple sclerosis—diminished afferent input has been linked to instability and fall risk [21,22]. Conversely, plantar sensory stimulation, including vibration or textured insoles, has been shown to improve postural control and balance in both healthy and clinical populations [23,24]. Recent studies also show that plantar vibration enhances ankle proprioceptive acuity, supporting the interaction between cutaneous and joint sensory systems [25]. Impaired plantar input may further exacerbate postural instability and injury risk for individuals with GJH, who already display deficits in proprioception and tactile sensation [22,23]. Therefore, targeted plantar sensory training can potentially strengthen sensorimotor loops, enhance cortical reorganization, and restore functional stability, offering a low-cost, home-based strategy suitable for broader clinical use.

Accordingly, the present study aimed to investigate the effects of plantar sensory training on plantar sensation, proprioception, balance, and muscle strength in young adults with GJH. This study contributes to the growing body of literature on physiotherapeutic strategies for GJH by addressing a critical gap and evaluating a feasible, accessible, and potentially preventive intervention.

## 2. Materials and Methods

### 2.1. Participants

Participation was voluntary, and all participants provided written informed consent. The Erzurum Technical University Scientific Research and Publication Ethics Committee approved the study, which was conducted in accordance with the Declaration of Helsinki (Approval No: 16/07/28.12.2023). The reporting of this trial followed the CONSORT guidelines [26].

**Figure 1:**
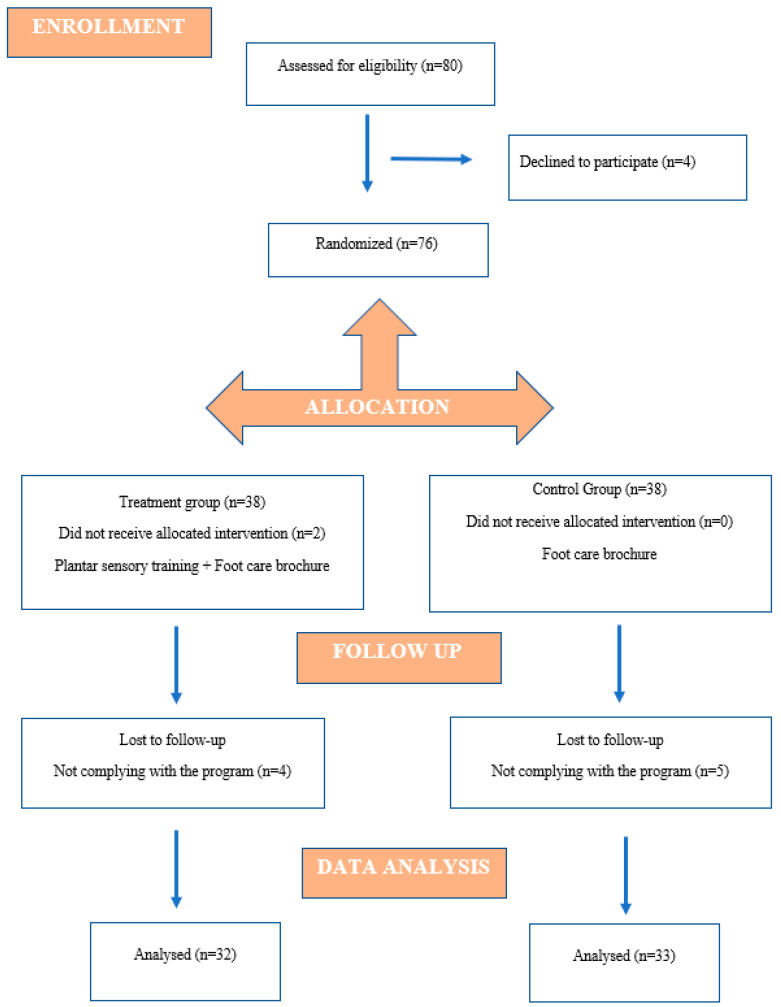
CONSORT flowchart showing participation in the study.

Asymptomatic individuals aged 18 to 25 with a Beighton score ≥5 and no systemic diseases were included. The Beighton score assesses joint hypermobility through goniometric flexibility measurements at the thumb, 5th metacarpal, elbow, knee, and spine joints [1]. The exclusion criteria included any upper or lower extremity injury within the past 3 months, a history of fracture or surgery within the previous year, pregnancy in the last year, a diagnosis of Ehlers–Danlos syndrome (EDS), hypermobile Ehlers–Danlos syndrome (hEDS), or hypermobility spectrum disorders (HSDs). The study design and participant flow are shown in the CONSORT flowchart (Figure 1).

Participants were randomized independently to treatment or control groups based on a computer-generated randomization list. The evaluator and statistician kept the randomization list confidential until data analysis was completed. Following the baseline assessment, the treatment group received a 2-week home program that included plantar sensory training and an educational foot care brochure, whereas the control group received only the brochure. Sensory tests (light touch, vibration, two-point discrimination, and proprioception), muscle strength assessments, and the Y balance test were performed before and after the intervention. All assessments were conducted by a single physiotherapist with 10 years of experience who was blinded to group allocation (RY).

### 2.2. Power Analysis

Before initiating participant recruitment, an a priori power analysis was performed via G*Power 3.1 to determine the minimum sample size required to detect a group × time interaction in a two-group repeated-measures design (pre- and postintervention) [27]. Given the study’s exploratory nature and the expectation of a pronounced intervention effect, particularly in domains such as proprioception and balance, which are directly targeted by plantar sensory stimulation, a large effect size of f = 0.408 was conservatively assumed. This assumption was based on the hypothesis that the targeted sensorimotor improvements would be both functionally meaningful and neurologically salient owing to the direct engagement of plantar mechanoreceptors. Under these conditions, with a significance level of 0.05 and a desired power of 0.90, the required sample size was estimated as *n* = 65. This sample size was therefore deemed sufficient to detect robust treatment effects in this population.

### 2.3. Outcome Measures

Demographic data were recorded for all participants. Beighton scoring was performed on five movements: dorsiflexion of the fifth metacarpal joint, thumb touching the forearm, hyperextension of the elbow and knee, and trunk flexion [1].

Physical activity levels were assessed using the International Physical Activity Questionnaire Short Form (IPAQ-SF). Responses were converted into metabolic equivalent (MET) minutes per week, and participants were classified according to the IPAQ scoring protocol: inactive (<600 MET-min/week), minimally active (600–2999 MET-min/week), and very active (≥3000 MET-min/week) [28]. These categories were used to describe baseline physical activity distribution in the sample and to compare group equivalence at baseline.

All sensory assessments, including light touch, vibration, two-point discrimination, and joint position sense, were performed in the standardized supine position with the participants’ feet bare and unclothed. This ensured consistency across all sensory testing procedures. Tests for light touch, vibration, and two-point discrimination were applied at the 1st metatarsal head, 5th metatarsal head, and heel [29]. The obtained data were presented separately for the right and left feet.

Light Touch Sensation: This was assessed via the Semmes–Weinstein Monofilament (SWM) test kit (North Coast Medical, San Jose, CA, USA). The monofilament with the smallest value (1.65) was used first. The monofilaments were in contact with the test points for 1.0–1.5 s. When the subject correctly felt the stimulus on one of three trials, the filament representing a certain force was noted as the subject’s score [30].

Two-Point Discrimination: A discriminator (Baseline 1, White Plains, NY, USA) was used to evaluate two-point discrimination. The evaluations were made by applying equal pressure at both ends of the discriminator simultaneously, and both ends were touched to each test point 3 times. If the individual felt two as one or reported that they could not distinguish them in the three-time evaluation, this distance was recorded as the two-point discrimination score [31].

Vibration: A 128 Hz tuning fork (Elcon1 Medical Instruments, Tuttlingen, Germany) was used to assess vibration sense. The tuning fork was applied vertically to the skin surface to be tested. The time was recorded with a stopwatch. The individual was asked to say “done” when they did not feel the vibration, and the time was recorded in seconds. The average of the three trials was recorded in seconds [32].

Proprioception: The joint position sense (JPS) test was performed using a digital goniometer device for proprioception assessment (Baseline Evaluation Instrument^®^, Fabrication Enterprises, Inc., Elmsford, NY, USA). The ankle JPS target angle was 15° for dorsiflexion and 25° for plantar flexion. The participant was placed in the supine position for the ankle JPS test. In the JPS tests, the joint was brought to the target angle while the subject’s eyes were closed, and the position was requested to be perceived after a 10 s wait. Then, the target angle was returned to the neutral position, repeated with the eyes closed, and the measurement was performed with a digital goniometer. The difference between the target angle and the participant’s result during the test was considered the error score. Three measurements were made for all target angles, and the arithmetic mean of the differences was calculated [33].

Muscle strength: A handheld dynamometer (Lafayette Instrument, Lafayette, IN, USA) was used to determine the muscle strength of both lower extremities in the individuals. Hand-held dynamometry has been shown to provide valid and reliable measurements of lower limb strength when standardized positions are used [34]. The measurements were recorded in kg. The evaluation measured the muscle strength values of the hip flexors, knee extensors, ankle dorsiflexors, and plantar flexors. Hip flexors, knee extensors, and ankle dorsiflexors were evaluated while sitting on the edge of the bed. The strength of the ankle plantar flexors was measured by hanging the ankle from the treatment bed while the individual was in a supine position. The measurements were repeated 3 times for each region, and the average of the measurements was recorded [34].

Balance: Individuals’ dynamic balance was assessed using the Y Balance Test. The test procedures were designed on a flat surface, with an angle of 135° between the anterior and posterior directions, and an angle of 90° between the posteromedial and posterolateral directions. Three tape measures were positioned on the floor in the specified directions, with the starting point at the center. The individuals began the test by placing their feet exactly in the center. To learn the test, the participants were given three trials to reach in the anterior, posteromedial, and posterolateral directions. After the test was administered, the individuals were asked to move 3 times in each direction within the framework of the instructions, and the average reach distances of the individuals for each direction were recorded in centimeters [35].

### 2.4. Intervention

Treatment Group: Following the initial assessment, participants in the treatment group received a home program consisting of exercises and suggestions. In line with the Consensus on Exercise Reporting Template (CERT) recommendations [36], the intervention protocol for the experimental group is described in detail as follows:

- Materials: Participants in the treatment group were provided with a spiky proprioceptive ball and three carpet pieces with different hardness levels to ensure program standardization. Additionally, a 500 mL water bottle and a chair were used for specific exercises. Warm water and moisturizing cream were recommended for foot preparation.
- Provider: A physiotherapist with 10 years of clinical experience initially demonstrated and instructed the intervention program. Participants also received written guidelines and an informative brochure to support the home application.
- Delivery: The program was delivered as a home-based, individually performed exercise protocol. Participants applied the exercises once daily, independently, after receiving initial training. Standardization was supported through a face-to-face demonstration session held before the program began.
- Location: The intervention was conducted in the participants’ home environment.
- Dosage (Frequency, Intensity, Duration)
  - Duration of program: 2 weeks.
  - Frequency: 1 session per day.
  - Session length: Approximately 20–25 min.
  - Exercises included: Washing the feet with warm water and applying moisturizing cream, Static stretching of the gastro-soleus and plantar fascia, Picking up a sheet with the toes, Rolling a 500 mL water bottle back and forth under the sole, Distinguishing three different textured surfaces without visual cues, Circular rolling of the spiky ball on the sole.
- Tailoring (Individualization/Progression): The same exercise set was given to all participants. However, minor adjustments were allowed for difficulty, such as modifying the surface hardness used during sensory discrimination tasks according to participants’ tolerance levels.
- Compliance/Adherence: The intervention was performed in the participants’ home environment. To ensure standardization, a physiotherapist provided a face-to-face demonstration at the start of the program, accompanied by a brochure and written instructions. Each participant was given an exercise log to record daily adherence, and reminder messages were sent once per day.

Control Group: A foot care brochure was distributed to the control group after the initial assessment, and participants were encouraged to read and carefully apply the recommendations. The brochures emphasized the importance of healthy feet, provided basic foot care techniques, offered general foot care recommendations, offered tips for selecting appropriate footwear, and provided information on when to consult a doctor [37].

### 2.5. Statistical Analysis

The statistical analysis proceeded in three stages: exploratory data analysis (EDA), assumption testing, and inferential modeling. First, EDA was conducted to characterize the distributional properties and temporal trends of the primary outcome variables. For each variable, descriptive statistics—including medians and interquartile ranges (IQRs)—were reported separately by group (control vs. treatment) and time point (pre vs. post). This preliminary step provided an empirical foundation for evaluating directional changes across conditions and informed subsequent modeling choices. Demographic characteristics were summarized and analyzed to assess baseline comparability between the control and treatment groups. Continuous variables are presented as medians and IQRs, and group differences were evaluated via the Mann–Whitney U test due to violations of normality. Categorical variables (e.g., sex and IPAQ score) were compared via Fisher’s exact test, which is appropriate for small and imbalanced samples. The Shapiro–Wilk test formally assessed the distributional assumptions of all outcome variables. None of the variables met the assumption of normality. Although Box–Cox transformations were applied, the transformed data still deviated from normality, precluding the use of traditional parametric methods. As part of EDA, box plots were constructed to visualize the distributional characteristics and temporal changes in composite outcome variables across groups. These plots enabled a preliminary examination of shifts in central tendency and variability between pre- and post-intervention measurements within each group. Composite scores were calculated as the arithmetic mean of their respective subdimensions to represent sensorimotor domains parsimoniously.

Given the nonnormality and repeated-measures design, a nonparametric rank-based mixed ANOVA was employed to evaluate the effects of group, time, and the group × time interaction. This approach offers robustness against violations of normality and is particularly well-suited for detecting differential temporal trajectories across groups, which is the central focus of this study. With a significance level of α = 0.05, all the statistical analyses were performed via the R programming language (version 4.4.2) [38].

## 3. Results

The results are presented in two stages: first, exploratory data analysis provides an overview of distributional characteristics and temporal patterns across groups; second, inferential statistics based on rank-based mixed ANOVA examine the main and interaction effects of group and time on the composite outcome variables.

### 3.1. Exploratory Data Analysis

Baseline comparisons were conducted to evaluate participants’ demographic and clinical characteristics across the control and treatment groups as part of the exploratory data analysis. Continuous variables were assessed for normality using the Shapiro–Wilk test. Due to violations of the normality assumption, nonparametric tests were applied to examine between-group differences. Appropriate tests (e.g., Fisher’s exact test) assessed group comparability for categorical variables. Subsequently, composite scores for the primary outcome variables were visualized via box plots to depict distributional characteristics and pre–post changes by group. In addition, summary tables reporting medians and interquartile ranges (IQRs) were generated for each outcome, offering a descriptive overview of temporal trends and variability.

Table 1 presents the baseline demographic and clinical characteristics of the participants in the treatment and control groups. No significant differences in age, weight, height, or Beighton score were detected between the treatment and control groups after appropriate statistical comparisons (*p* > 0.05). The sex distribution and IPAQ scores were comparable between the groups (*p* *** > 0.05).

Table 2 presents the median and interquartile range (IQR) values for all outcome variables at the pre- and postintervention time points, stratified by group.

For statistical analysis and graphical representation, composite scores were generated for each primary outcome variable (i.e., light touch, two-point discrimination, vibration, muscle strength, balance, and proprioception). These composite scores, visualized in the box plots presented here, represent the arithmetic mean of multiple subdimensions that assess distinct but related facets of each domain. This aggregation strategy was employed to reduce dimensionality, enhance interpretability, and ensure a theoretically coherent representation of the underlying constructs.

**Figure 2:**
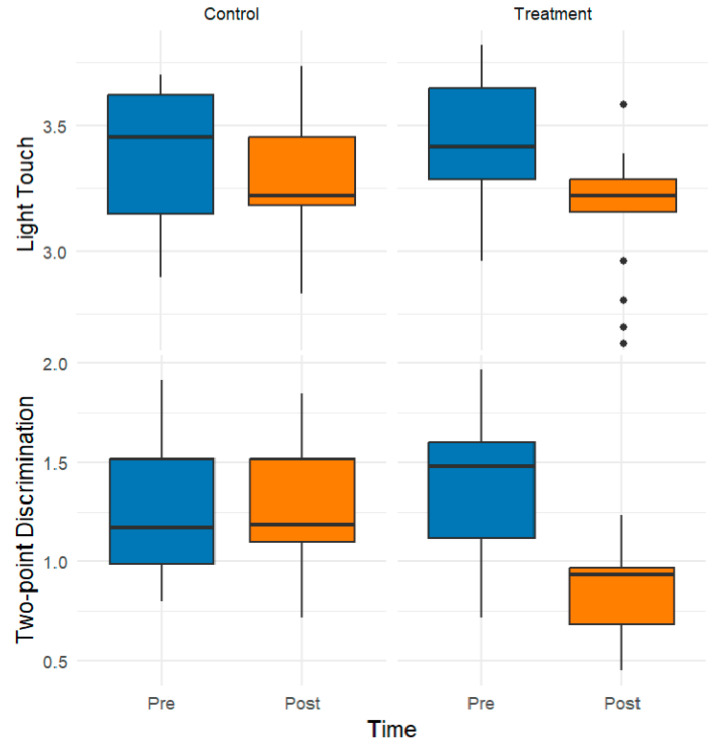
Effects of the Intervention on Light Touch and Two-Point Discrimination: Pre- and Postintervention Comparisons across the Control and Treatment Groups. The black circles indicate outlier data points.

Figure 2 shows the pre- and postintervention composite scores, which were derived as the means of their light touch and two-point discrimination subdimensions. Compared with the preintervention group, the treatment group exhibited notable postintervention improvements in light touch sensitivity and two-point discrimination. In particular, the median values decreased substantially in the treatment group, indicating enhanced sensory performance, whereas the control group showed minimal or no change.

**Figure 3:**
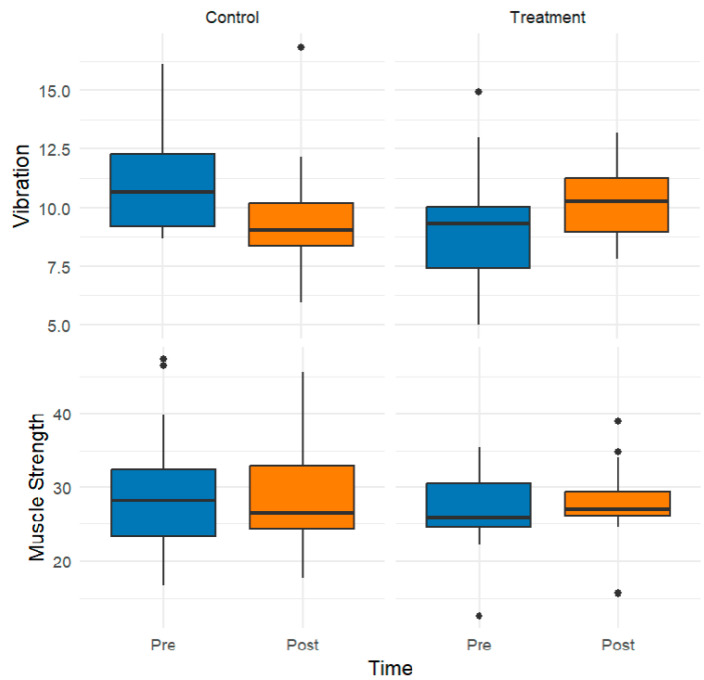
Effects of the Intervention on Vibration and Muscle Strength: Pre- and Postintervention Comparisons across the Control and Treatment Groups. The black circles indicate outlier data points.

Figure 3 illustrates the groupwise distributions of vibration sensation and muscle strength, represented as composite scores derived from the means of their respective subdimensions. Following the intervention, the treatment group demonstrated modest increases in the median values for both outcomes, indicating potential improvements in neurosensory perception and muscular performance. In contrast, the control group presented stable or slightly diminished scores over time.

**Figure 4:**
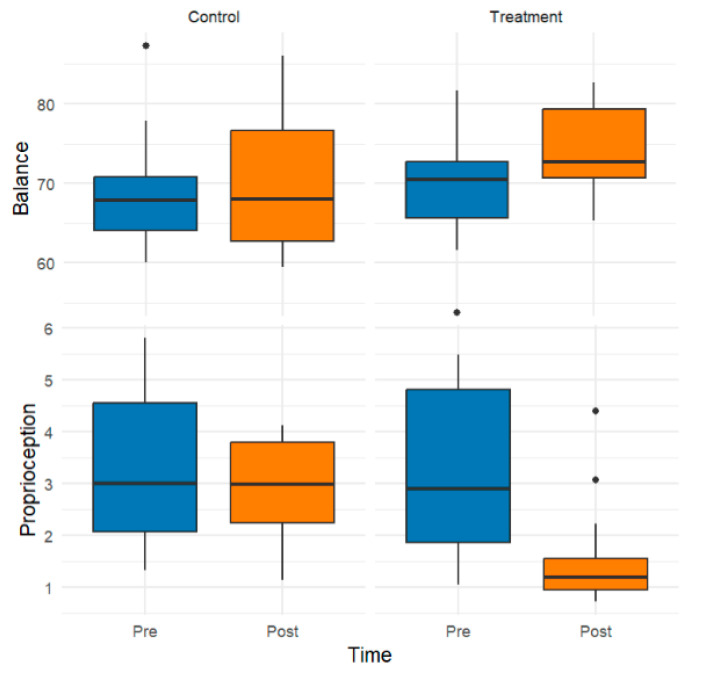
Effect of the intervention on balance and proprioception: Pre-post comparison across the control and treatment groups. The black circles indicate outlier data points.

Figure 4 presents the distributions of composite scores for both groups’ balance and proprioception across the pre- and postintervention assessments. These scores were computed as the arithmetic mean of multiple task-specific subdimensions designed to capture distinct postural control and sensory–motor coordination elements. Following the intervention, the treatment group exhibited substantial improvements, reflected by increased balance scores and a sense of proprioception. In contrast, the control group showed no meaningful change over time.

### 3.2. Inferential Findings from Rank-Based Mixed ANOVA

Following the preliminary data exploration, rank-based mixed ANOVAs were conducted to examine the effects of group (Control vs. Treatment), time (Pre vs. Post), and their interaction on each composite outcome variable. Given the nonnormal distribution of dependent variables, a rank-based approach was adopted to ensure the robustness of the inference while accommodating both within-subject (time) and between-subject (group) factors. The results of these analyses are presented below for each outcome domain, with particular attention given to statistically significant main and interaction effects (Table 3).

A significant main effect of time was observed for most outcome variables, indicating overall improvement from pre- to post-assessment across participants. Notably, the most compelling results emerged from significant group-by-time interaction effects observed across multiple sensorimotor domains. These interaction effects indicate that the pattern and magnitude of change differed significantly between groups, with the treatment group exhibiting greater functional gains. As such, the group × time interaction is captured.

## 4. Discussion

This study is the first randomized controlled trial to examine the effects of plantar sensory training on sensorimotor organization in individuals with GJH. The findings demonstrated that targeted somatosensory stimulation significantly enhanced tactile sensitivity, proprioception, and dynamic balance.

Interventions for individuals with GJH are limited in the literature. A systematic review by Scheper et al. revealed that no studies have examined specific treatment approaches for GJH. In contrast, five studies in individuals with joint hypermobility syndrome reported only minor effects on pain and inconsistent effects on disability [39]. A recent study reported that a resistance training program applied to women with GJH had no significant impact on muscle strength, muscle mass, pain, or daily living activities [13]. On the other hand, a randomized controlled study conducted by Hornsby et al. in 2024 found that a Pilates program, applied to school-aged children with GJH under the guidance of a physiotherapist, resulted in a decrease in pain, an increase in muscle strength, and an improvement in health-related quality of life [14]. These findings suggest that effective intervention methods for individuals with GJH are still under investigation, and individualized approaches are necessary. Our current study aims to fill this gap in the literature by systematically evaluating the effects of somatosensory-based sensory training on sensorimotor organization components such as balance, proprioception, and tactile perception in individuals with GJH. Significant improvements were observed after the intervention, indicating that holistic approaches targeting motor performance and sensory integration can be practical in GJH.

When the study results regarding sensory parameters were evaluated, significant improvements in light touch, two-point discrimination, and vibration sensation were observed, suggesting that tactile inputs received from the sole may enhance sensorimotor integration. In individuals with GJH, sensory transmission and mechanoreceptor activity impairments can be observed due to the flexibility of the connective tissue [40,41]. In this context, sensory training may have contributed to the reorganization of the sensorimotor system by compensating for existing deficiencies. One possible mechanism is the increased activation of mechanoreceptors and improved cortical representation of the foot region, which contributes to better sensory-motor coupling. While the intervention duration was limited, the magnitude of sensory gains suggests that even brief programs may trigger neuroplastic changes.

Significant improvements in proprioceptive sense indicate that motor planning and postural control mechanisms, critical components of sensorimotor organization, are strengthened. Significant differences in ankle joint position sensory tests suggest that the central nervous system processes sensory information from peripheral proprioceptive receptors more accurately. Patel et al. reported that somatosensory training improved proprioception in dancers with GJH and nondancers in their study of young children with GJH [15]. Similarly, the plantar somatosensory training used in the current study may have increased proprioceptive awareness by targeting sensory deficits.

The improvements observed in balance performance show that sensory training is reflected in functional outcomes. According to the Y balance test results, the reaching distances increased. These results show that sensory training is not limited to passive sensory gains but also affects active motor responses. A study by Hijmans et al. revealed that vibratory stimulation at the plantar surface increased balance performance [42]. Viseux et al. emphasized the effect of plantar receptors on postural control; the home program-based intervention method used in this study provided similar gains [43].

Although improvements in muscle strength were observed in some muscle groups, the group-by-time interaction was not statistically significant for any individual muscle group. These findings suggest that plantar sensory training supports motor performance indirectly, rather than directly, by increasing muscle strength. Increased somatosensory inputs received from the plantar surface may facilitate motor unit activation by improving postural awareness and movement control; however, this process may not be sufficient to induce physiological changes, such as muscle hypertrophy or an increase in maximal strength. The literature suggests that progressive resistance training is necessary for a specific period to achieve strength development [44]. In this context, the two-week sensory training program may have only supported neural adaptations; however, it may have been insufficient for structural changes such as muscle hypertrophy. In line with these findings, the need to support the effect of somatosensory training on strength increase with longer-term and targeted strength training is an important point to consider in future studies.

Our results were compared with minimum detectable change (MDC) values reported in the literature to ensure that the changes were not due to measurement error. For light touch sensation assessed with the Semmes–Weinstein monofilament, Suda et al. reported MDC values between 1.28 and 2.06 filament steps [45], and the improvements in our study surpassed this threshold. Although a statistically significant improvement in plantar two-point discrimination was observed in our study, the magnitude of change remained below the MDC reported in previous studies. Zimney et al. [46] reported an MDC of ≈12.3 mm, and Suganuma et al. [47] calculated MDC95 values between 1.82 and 2.19 cm. Therefore, in our study, the observed change, while statistically significant, may not be interpreted as a clinically meaningful improvement beyond measurement error. For vibration sense assessed with the 128 Hz tuning fork, no directly reported MDC values are available in the literature. Therefore, although statistically significant, the results obtained in our study should be interpreted with caution, as they may not definitively represent clinically meaningful changes beyond measurement error. Notably, in plantar flexion, the reduction in joint position error (≈2–3°) exceeded the previously reported MDC95 of 1.90° for ankle proprioception [47]. This suggests that the improvements observed in this direction are not only statistically significant but also clinically meaningful, reflecting a true enhancement of proprioceptive acuity beyond measurement error. In the present study, the observed improvement in the posterolateral direction of the Y-Balance Test was approximately 8.3 cm, corresponding to 8.7–9.8% of the total distance, depending on limb length (85–95 cm). This exceeds the reported MDC95 for the posterolateral direction (8.08%) [48], indicating that the change is beyond measurement error. Furthermore, based on a distribution-based approach (0.5 SD) [49], this magnitude of change also surpasses the threshold for minimal clinically significant difference, suggesting that the observed improvement is both statistically significant and clinically meaningful.

This study is valuable in that it is the first randomized controlled trial to examine the effects of plantar sensory training on sensorimotor organization in individuals with GJH, but it has several limitations. First, the fact that the sample group used in the study consisted only of young, asymptomatic individuals between the ages of 18 and 25 limits the generalizability of the findings to wider age groups or symptomatic individuals with GJH. Additionally, the fact that most participants were female limited the comprehensive assessment of sensory and motor differences related to sex. Another limitation is that the intervention was conducted at home without direct supervision. Although exercise logs and daily reminder messages were used to encourage adherence, verifying whether participants consistently and accurately implemented the program was impossible. This may have made it difficult to control the application dose and standardization.

In summary, our findings support the integration of plantar somatosensory training as a complementary, accessible approach to improve sensorimotor function in young adults with GJH. The feasibility of home-based programs may increase patient adherence and accessibility, making them promising adjuncts to conventional therapies. However, these findings should be interpreted cautiously because they were obtained from a young, asymptomatic sample. Future studies are needed to determine whether similar benefits of plantar sensory training can be observed in symptomatic individuals, different age groups, or populations with comorbidities. Future research should explore the combination of sensory training with targeted strength and functional exercises, and evaluate the effects in diverse populations to optimize intervention strategies for individuals with GJH.

## 5. Conclusions

This randomized controlled trial demonstrated that a 2-week plantar sensory training program significantly improved tactile sensitivity, proprioception, and balance in young adults with GJH. These findings suggest that simple, home-based sensory stimulation targeting the plantar surface can effectively enhance key components of sensorimotor organization without the need for complex equipment or supervised sessions. Given its low cost, ease of implementation, and demonstrated benefits, plantar sensory training holds promise as a practical adjunct to conventional rehabilitation protocols for individuals with joint hypermobility. However, further research is needed to investigate the long-term effects and to evaluate its integration with strength and coordination training programs.

## Figures and Tables

**Table 1:** Comparison of demographic and clinical characteristics between the treatment and control groups.

Variable	Treatment Median (IQR)	Control Median (IQR)	*p* *	*p* **
Age (years)	20.00 (2.00)	20.00 (1.25)	<0.001	0.633
Weight (kg)	65.00 (16.00)	55.00 (20.00)	<0.001	0.137
Height (cm)	164.00 (8.00)	164.00 (5.00)	<0.001	0.859
Beighton Score	8.50 (2.00)	8.00 (0.75)	<0.001	0.688
	*n* (%)	*n* (%)	Total	*p* ***
Gender				0.105
Female	27 (84.37)	32 (96.97)	59 (90.76)	
Male	5 (15.62)	1 (3.03)	6 (9.23)	
IPAQ Level				
Inactive	25 (78.13)	27 (81.82)	52 (80)	
Minimally active	4 (12.5)	6 (18.18)	10 (15.38)	0.209
Very active	3 (9.38)	0 (0)	3 (4.62)	

*p* *: Shapiro–Wilk test, *p* **: Mann–Whitney U test, *p* ***: Chi-square test, Fisher’s exact test, IQR: Interquartile range. IPAQ: International Physical Activity Questionnaire Short Form.

**Table 2:** Descriptive Statistics Before and After Treatment and for the Control Group as the Median (IQR).

Muscle Strength (kg)		Control		Treatment	
		Pre	Post	Pre	Post
Knee extension	R	35.00 (8.77)	32.40 (13.10)	27.50 (12.70)	36.60 (4.50)
	L	34.55 (16.63)	31.50 (15.90)	32.00 (7.30)	33.30 (9.70)
Hip flexion	R	30.00 (7.70)	28.00 (8.90)	28.80 (13.10)	27.00 (8.10)
	L	27.70 (7.80)	24.50 (6.40)	28.10 (6.90)	25.00 (14.30)
Plantar flexion	R	28.80 (11.90)	26.30 (14.80)	25.60 (5.60)	28.40 (7.40)
	L	30.60 (3.20)	31.60 (13.10)	27.60 (2.50)	21.40 (11.60)
Dorsiflexion	R	18.00 (12.20)	22.50 (5.00)	19.20 (8.30)	22.50 (6.60)
	L	16.40 (13.70)	20.20 (10.30)	18.90 (7.50)	24.00 (3.20)
Light touch					
First metatarsal head	R	3.20 (0.40)	3.60 (0.40)	3.20 (0.00)	3.60 (0.40)
	L	3.20 (0.40)	3.20 (0.40)	3.20 (0.80)	3.60 (0.60)
Fifth metatarsal head	R	3.20 (0.40)	3.60 (0.40)	3.60 (0.40)	3.60 (0.40)
	L	3.20 (0.50)	3.40 (0.40)	3.60 (0.40)	3.20 (0.00)
Heel	R	3.60 (0.60)	3.20 (0.10)	3.60 (0.60)	3.20 (0.40)
	L	3.20 (0.40)	3.20 (0.40)	3.60 (0.60)	3.60 (0.60)
Two-point discrimination (mm)					
First metatarsal head	R	1.20 (0.40)	1.00 (0.60)	1.40 (0.40)	0.80 (0.40)
	L	1.10 (0.60)	1.10 (0.30)	1.40 (0.30)	0.90 (0.40)
Fifth metatarsal head	R	1.30 (0.50)	1.30 (0.40)	1.40 (0.60)	0.80 (0.40)
	L	1.00 (0.50)	1.20 (0.60)	1.30 (0.90)	0.80 (0.40)
Heel	R	1.20 (0.20)	1.10 (0.40)	1.50 (0.60)	1.00 (0.20)
	L	1.20 (0.30)	1.20 (0.60)	1.50 (0.20)	1.00 (0.60)
Vibration (sec)					
First metatarsal head	R	9.60 (4.10)	10.00 (3.40)	10.60 (1.80)	9.80 (4.10)
	L	9.40 (5.30)	13.00 (3.20)	9.70 (3.40)	8.90 (2.30)
Fifth metatarsal head	R	8.10 (2.60)	9.50 (2.30)	10.30 (2.80)	10.60 (2.40)
	L	9.40 (5.30)	13.00 (3.20)	9.70 (3.40)	8.90 (2.30)
Heel	R	9.00 (3.10)	12.10 (2.20)	10.60 (1.90)	8.00 (3.50)
	L	9.70 (3.10)	9.80 (2.20)	9.60 (1.90)	9.20 (3.50)
Proprioception (◦)					
Dorsiflexion	R	2.10 (1.90)	2.30 (1.40)	2.60 (3.70)	1.10 (1.70)
	L	2.00 (2.10)	2.30 (2.30)	1.10 (4.00)	1.00 (0.10)
Plantar flexion	R	2.30 (1.00)	4.00 (2.30)	4.30 (5.30)	1.30 (0.60)
	L	3.00 (3.30)	5.00 (3.70)	3.30 (2.00)	1.60 (1.30)
Balance (cm)					
Anterior		61.60 (4.90)	65.80 (11.00)	66.00 (12.70)	70.00 (7.40)
Posterolateral		74.60 (8.72)	70.50 (11.70)	72.00 (3.30)	80.30 (12.00)
Posteromedial		71.00 (12.25)	74.00 (15.47)	72.00 (20.00)	72.00 (8.00)

R: right, L: left.

**Table 3:** Overview of the statistical significance of time, group, and interaction effects across variables.

Variable		*p* (Time)	*p* (Group)	*p* (Group × Time)
Muscle Strength (kg)				
Knee extension	R	0.051	0.583	0.550
	L	0.602	0.810	0.697
Hip flexion	R	**<0.01**	0.316	0.175
	L	**0.021**	0.545	0.394
Plantar flexion	R	0.767	0.442	0.961
	L	**0.030**	**0.003**	0.831
Dorsiflexion	R	0.102	0.847	0.101
	L	**0.001**	0.151	0.584
Light touch				
First metatarsal head	R	**<0.01**	0.964	**<0.01**
	L	**<0.01**	0.960	**0.002**
Fifth metatarsal head	R	**<0.01**	**0.023**	0.059
	L	0.062	0.572	**<0.01**
Heel	R	**<0.01**	0.451	0.501
	L	**<0.01**	0.694	0.902
Two-point discrimination (mm)				
First metatarsal head	R	**<0.01**	**0.008**	**<0.01**
	L	**<0.01**	**0.010**	**<0.01**
Fifth metatarsal head	R	**<0.01**	0.072	**<0.01**
	L	**<0.01**	0.080	**0.001**
Heel	R	**<0.01**	0.424	**<0.01**
	L	**<0.01**	0.399	**<0.01**
Vibration (sec)				
First metatarsal head	R	0.263	0.821	**<0.01**
	L	**0.009**	**0.039**	**<0.01**
Fifth metatarsal head	R	**0.048**	0.091	**0.023**
	L	0.099	0.382	**0.025**
Heel	R	0.410	0.121	**<0.01**
	L	0.326	0.427	**0.028**
Proprioception (◦)				
Dorsiflexion	R	**<0.01**	0.125	**<0.01**
	L	**0.002**	**<0.01**	0.275
Plantar flexion	R	**<0.01**	**0.012**	**0.007**
	L	**<0.01**	**0.005**	**0.016**
Balance				
Anterior		**<0.01**	**0.040**	0.160
Posterolateral		**<0.01**	0.437	**<0.01**
Posteromedial		**0.016**	0.377	0.069

R: right, L: left. This table highlights statistically significant results (*p* < 0.05) in bold.

## Data Availability

The datasets used and/or analyzed during the current study are available from the corresponding author upon reasonable request.

## Abbreviations

The following abbreviations are used in this manuscript:

GJH	Generalized Joint Hypermobility
EDS	Ehlers–Danlos Syndrome
hEDS	Hypermobile Ehlers–Danlos Syndrome
HSDs	Hypermobility Spectrum Disorders
CONSORT	Consolidated Standards of Reporting Trials
IPAQ	International Physical Activity Questionnaire
SWM	Semmes–Weinstein Monofilament
JPS	Joint Position Sense
EDA	Exploratory Data Analysis
ANOVA	Analysis of Variance
TUBITAK	Türkiye Bilimsel ve Teknolojik Araştırma Kurumu
